# Addition of V-Stage to Conventional TNM Staging to Create the TNVM Staging System for Accurate Prediction of Prognosis in Colon Cancer: A Multi-Institutional Retrospective Cohort Study

**DOI:** 10.3390/biomedicines9080888

**Published:** 2021-07-25

**Authors:** Jung Hoon Bae, Ji Hoon Kim, Jaeim Lee, Bong-Hyeon Kye, Sang Chul Lee, In Kyu Lee, Won Kyung Kang, Hyeon-Min Cho, Yoon Suk Lee

**Affiliations:** 1 Department of Surgery, Division of Colorectal Surgery, Seoul St. Mary’s Hospital, College of Medicine, The Catholic University of Korea, Seoul 06591, Korea; eysi0815@catholic.ac.kr (J.H.B.); cmcgslee@catholic.ac.kr (I.K.L.); 2 Department of Surgery, Division of Colorectal Surgery, Incheon St. Mary’s Hospital, College of Medicine, The Catholic University of Korea, Incheon 21431, Korea; samryong@catholic.ac.kr; 3 Department of Surgery, Division of Colorectal Surgery, Uijeongbu St. Mary’s Hospital, College of Medicine, The Catholic University of Korea, Uijeongbu-si 11765, Korea; lji96@catholic.ac.kr; 4 Department of Surgery, Division of Colorectal Surgery, St. Vincent’s Hospital, College of Medicine, The Catholic University of Korea, Suwon-si 16247, Korea; ggbong@catholic.ac.kr (B.-H.K.); hmcho@catholic.ac.kr (H.-M.C.); 5 Department of Surgery, Division of Colorectal Surgery, Daejeon St. Mary’s Hospital, College of Medicine, The Catholic University of Korea, Daejeon 34943, Korea; zambo9@catholic.ac.kr; 6 Department of Surgery, Division of Colorectal Surgery, Yeouido St. Mary’s Hospital, College of Medicine, The Catholic University of Korea, Seoul 07345, Korea; wonkkang@catholic.ac.kr

**Keywords:** colonic neoplasm, vascular invasion, lymph node metastasis, TNVM staging

## Abstract

We evaluated the prognostic impact of vascular invasion (VI) compared with nodal (N) stage and developed a new staging system including VI in colon cancer. Patients who underwent curative resection with stage II-III colon cancer were assigned to VI and non-VI groups; the latter was subclassified as N0, N1, and N2; a new TNVM staging was devised by adding the V-stage. Among the 2243 study participants, the VI group independently showed worse oncological outcomes than the N1 group (disease-free survival (DFS), hazard-ratio (HR) 1.704, 1.267–2.291; overall survival (OS), HR 2.301, 1.582–3.348). The 5-year DFS in the VI group was 63.4% [N1b (74.6%), *p* = 0.003; N2a (69.7%), *p* = 0.126; and N2b (56.8%), *p* = 0.276], and the 5-year OS was 76.6% [N1b (84.9%), *p* = 0.004; N2a (83.0%), *p* = 0.047; and N2b (76.1%), *p* = 0.906]. Thus, we considered VI as N2a in TNVM staging; 78 patients (3.5%) underwent upstaging. The 5-year OS rates of stage IIB and IIC increased from 88.6% and 65.9% in TNM staging to 90.5% and 85.7% in TNVM staging, respectively. In stage II–III colon cancer, VI had a similar prognostic impact as the N2 stage without VI. The incorporation of the V-stage into the conventional TNM staging facilitates better prediction of prognosis.

## 1. Introduction

Distant metastasis is the most common cause of death in patients with colon cancer [1,2]. In the management of colon cancer, it is very important to recognize the risk factors for distant metastasis and to accordingly establish appropriate treatment strategies [3,4]. Many efforts have been made to identify the risk factors beyond the tumor, node, metastasis (TNM) classification [4,5] and, consequently, poorly differentiated tumors, positive margin involvement, examination of <12 lymph nodes (LN), obstruction, perforation, perineural invasion, lymphatic invasion, and vascular invasion (VI) have been considered to be risk factors for distant metastasis [6,7]. A rapid paradigm shift to a more personalized treatment approach for colon cancer was reported recently [8,9]. However, in clinical practice, the treatment plan is mainly determined on the basis of the traditional TNM staging system. Most guidelines recommend adjuvant chemotherapy after curative resection for stage III colon cancer with lymph node metastasis (LNM). In contrast, in stage II colon cancer without LNM, even with high-risk factors, adjuvant chemotherapy is considered an optional treatment modality after curative surgery [6,7].

The main metastatic pathways in colon cancer are the lymphatic and vascular channels [10,11,12,13]. In TNM staging, T stage is the depth of invasion of the primary tumor. The presence of LNM within the lymphatic system, which is one of the metastatic pathways, is considered to indicate nodal (N) stage; and M stage is determined by distant metastasis [14]. However, the TNM staging system does not comprise factors related to the vascular system, which is another metastatic pathway. Many recent studies have reported a poor prognostic impact of VI [15,16,17]. One study showed that cancer-specific survival was worse in stage II cancer with VI than in stage III disease without VI [15]. Nonetheless, no study has attempted to incorporate VI into the TNM staging system.

This study was conducted with an aim to ascertain the prognostic relevance of VI in colon cancer. The primary objective was to evaluate the prognostic impact of VI, compared with the N stage, in stage II–III colon cancer after curative surgery. The secondary objective was to develop a novel staging system that would include VI, based on its prognostic impact.

## 2. Materials and Methods

### 2.1. Patients and Data Collection

This retrospective multicenter study involved a chart review of patients who underwent curative surgery for stage II–III colon cancer between January 2008 and December 2015 at six hospitals affiliated with the College of Medicine, The Catholic University of Korea and were considered eligible for study inclusion (*n* = 2309). Patients with missing data on pathologic results (*n* = 24), those who had a recurrence within 90 days after surgery (*n* = 23), and those who died within 30 days after surgery for any reason were excluded (*n* = 19). Finally, this study included 2243 patients with stage II–III colon cancer during the study period from different study centers: Seoul St. Mary’s Hospital (*n* = 623), Yeouido St. Mary’s Hospital (*n* = 279), Uijeongbu St. Mary’s Hospital (*n* = 176), Incheon St. Mary’s Hospital (*n* = 739), St. Vincent’s Hospital (*n* = 386), and Daejeon St. Mary’s Hospital (*n* = 40). 

Patient data, including the demographic and clinicopathological characteristics, recurrence, and survival records, were collected from each hospital’s colon cancer patient registry. The right colon was defined as including the cecum, ascending colon, hepatic flexure colon, and transverse colon, whereas the left colon was defined as comprising the splenic flexure colon, descending colon, sigmoid colon, and recto-sigmoid colon above the peritoneal reflection. The pathologic staging was classified in accordance with the Seventh American Joint Committee on Cancer (AJCC) TNM Classification System [14]. In addition, pathological data, such as the histological grade, VI, lymphatic invasion, and perineural invasion, were collected as potential prognostic factors. A well-differentiated or moderately differentiated adenocarcinoma was defined as a favorable histological grade, whereas poorly differentiated adenocarcinoma, mucinous carcinoma, or signet ring cell carcinoma were graded as a poor histological grade.

The presence of lymphovascular invasion was assessed on hematoxylin and eosin (H&E)-stained slides. According to the current pathology practice guidelines [18], when the tumor cells involve small vessels with an unequivocal endothelial lining, such as lymphatics, capillaries, and postcapillary venules, it was considered lymphatic (small vessel) invasion. In contrast, when carcinoma was present in vessels with an identifiable thick smooth muscle layer or elastic lamina, this was considered vascular (large vessel) invasion.

This study was approved by the Institutional Review Board of the Ethics Committee of the College of Medicine, The Catholic University of Korea (XC20RADI0183). Informed consent was obtained from all patients for study participation, and the records were anonymized and de-identified prior to analysis.

### 2.2. Study Design

The study participants were divided into two groups based on the presence of VI (non-VI and VI groups), regardless of the TNM stage. Subsequently, we subdivided the non-VI group into N0, N1, and N2 subgroups based on the N stage. Among the patients in the non-VI group, patients with N0 stage (no regional LNM), N1 stage (metastasis in 1–3 regional LNs), and N2 stage (metastasis in ≥4 regional LNs) were assigned to the N0, N1, and N2 groups, respectively. Finally, we compared the oncological outcomes between groups N0, N1, N2, and VI. All surgeries were performed by colorectal surgeons who were certified in the subspecialty of colorectal surgery by the Korean Surgical Society.

Based on the results of the comparative evaluation of the prognostic impact of VI with that of each N stage without VI, a new TNV staging system was devised by including the V stage (which indicates the presence of VI) into the traditional TN staging system. The V0 and V1 stages were defined as the absence and presence of VI, respectively. Next, by comparing the disease-free survival (DFS) and overall survival (OS) of each sub-stage in the TN staging and the new TNV staging, respectively, we evaluated the associations between each staging system and prognosis in stage II–III colon cancer.

### 2.3. Outcomes

The primary outcomes were DFS and OS. DFS was defined as the interval between the date of surgery and either the date that recurrence was detected on radiologic or pathologic examination or the last follow-up date in patients without recurrence. OS was calculated from the date of surgery to the date of death or the last follow-up. The follow-up data were censored in December 2020

### 2.4. Statistical Analysis

The chi-square or Fisher’s exact test was used to compare categorical variables. Logistic regression analysis was used to determine independent factors that were related to VI. The rates of DFS and OS were calculated using the Kaplan–Meier method, and the survival curves were compared using the log-rank test. The univariate prognostic significance of the variables was determined using the Cox proportional hazard model. Variables that were significantly associated with the survival rate on univariate analysis were subsequently included in the multivariate analysis that was performed using the Cox multiple regression model. Hazard ratios (HRs) with corresponding 95% confidence intervals (CIs) were estimated. *p*-values less than 0.05 were deemed to indicate statistical significance. All statistical analyses were performed using SPSS version 25.0 for Windows (IBM Corp., Armonk, NY, USA).

## 3. Results

### 3.1. Baseline Characteristics

A total of 2243 patients with stage II–III colon cancer were included in this study. The overall median follow-up duration was 54 months (interquartile range, 30–72 months), the mean patient age was 64.0 ± 12.2 years, and the male-to-female ratio was 1.23:1. Tumors were located in the right and left colon in 848 (37.8%) and 1395 (62.2%) patients, respectively. Surgeries were performed via a laparoscopic and conventional open approach in 1412 (63.0%) and 831 (37.0%) patients, respectively. There were 1045 (46.6%) and 1198 (53.4%) patients with stage II and III disease, respectively. Adjuvant chemotherapy was administered to 1772 patients (79.0%), and recurrence occurred in 420 patients (18.7%) during the study period.

In this study, VI was detected in 225 patients (10.0%). The stratification of the baseline patient characteristics based on the presence of VI is shown in Table 1. Patient characteristics, such as age, sex, American Society of Anesthesiologist (ASA) score, tumor location, surgical approach, adequate LN harvest number ( ≥ 12), and adjuvant chemotherapy, did not significantly differ between the non-VI and VI groups. Patients with VI had significantly higher rates of T4 tumor (*p* < 0.001), N2 stage (*p* < 0.001), stage III disease (*p* < 0.001), poor histological grade (*p* = 0.031), lymphatic invasion (*p* < 0.001), and perineural invasion (*p* < 0.001) than those without VI. The recurrence rate was significantly higher in patients with VI than in those without VI (31.6% vs. 17.3%, *p* < 0.001).

### 3.2. Baseline Patient Characteristics According to the Study Groups

Of the 2243 patients who were enrolled in this study, 987 (44.0%), 696 (31.0%), 335 (14.9%), and 225 (10.0%) were in the N0, N1, N2, and VI groups, respectively. Table 2 summarizes the characteristics according to these study groups, and there were significant differences in the rates of T4 tumor, adequate LN harvest number, histological grade, lymphatic and perineural invasion, adjuvant chemotherapy, and recurrence (*p* < 0.001 for all) between the four groups. Comparison of the N2 and VI groups showed that the rate of lymphatic invasion (*p* = 0.032) was higher in the patients in the N2 group than in the VI group. In contrast, the rates of T4 tumor (*p* = 0.003) and inadequate LN harvest (*p* = 0.036) were higher among the patients in the VI group than among those in the N2 group. The recurrence rates during the study period were 10.8%, 19.5%, 31.6%, and 31.6% in the N0, N1, N2, and VI groups, respectively.

T4 tumor (*p* < 0.001), N2 stage (*p* < 0.001), lymphatic invasion (*p* = 0.007), and perineural invasion (*p* = 0.001) were independently associated with the presence of VI in multiple logistic regression analysis (Table S1).

### 3.3. Univariate and Multivariate Analyses

The outcomes of the univariate and multivariate analyses that were conducted to identify significant prognostic factors for DFS and OS are shown in Table 3 and Table 4, respectively. We placed the N1 group as a reference in the Cox multiple regression model to compare the N stage and VI groups. In multivariate analysis, older age (≥ 65 years; HR, 1.260; 95% CI, 1.039–1.527), conventional approach (HR, 1.307; 95% CI, 1.073–1.593), T4 tumor (HR, 1.920; 95% CI 1.511–2.439), LN harvest number less than 12 (HR, 1.453; 95% CI, 1.132–1.864), and perineural invasion (HR, 1.259; 95% CI, 1.015–1.561) were independent prognostic factors for poor DFS. Patients in the N0 group (HR, 0.543; 95% CI, 0.407–0.722) had better prognosis, and those in the N2 (HR, 1.738; 95% CI, 1.343–2.251) and VI (HR, 1.704; 95% CI, 1.267–2.291) groups had worse prognosis in terms of DFS.

With regard to the OS, male sex (HR, 1.319; 95% CI 1.026–1.696), ASA Score ≥3 (HR, 2.531; 95% CI, 1.774–3.611), conventional approach (HR, 1.569; 95% CI 1.225–2.011), T4 tumor (HR, 2.110; 95% CI, 1.567–2.842), LN harvest number less than 12 (HR, 1.588; 95% CI 1.177–2.142), poor histological grade (HR, 1.577; 95% CI, 1.095–2.271), and no adjuvant chemotherapy (HR, 2.268; 95% CI, 1.699–3.027) were independently associated with poor prognosis in multivariate analysis. Patients in the N2 (HR, 1.949; 95% CI, 1.366–2.779) and VI (HR, 2.301; 95% CI, 1.582–3.348) groups had worse prognosis in terms of OS. There were no significant OS differences between patients in group N0 and N1.

### 3.4. Recurrence and Survival Outcome according to the N Stage and VI

The 5-year DFS and OS rates were 78.5% and 87.2%, respectively, in the entire study population. We tried to determine the prognostic impact of VI compared with each N stage. Patients in the non-VI group were subdivided into five categories on the basis of the AJCC cancer staging criteria (N0: no regional LNM; N1a: 1 regional LN metastasis; N1b: 2–3 metastases; N2a:4–6 metastases; and N2b: ≥7 metastases). 

**Figure 1:**
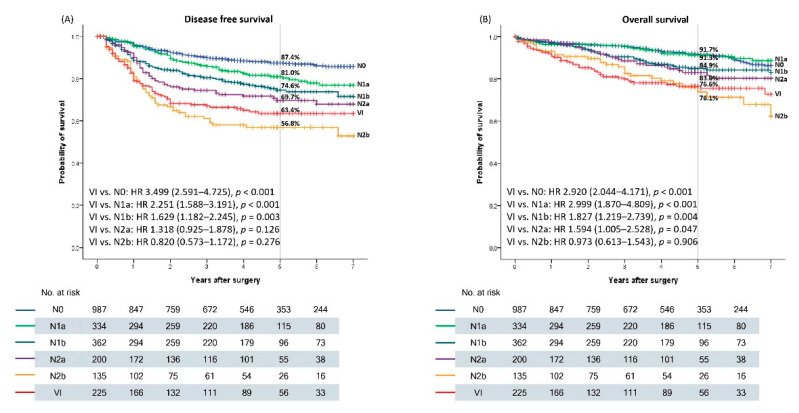
Kaplan–Meier curve according to the study groups indicating (**A**) disease-free survival and (**B**) overall survival.

The Kaplan–Meier curves for DFS and OS are shown for each N stage and VI in Figure 1. The 5-year DFS rates were 87.4% for N0, 81.0% for N1a, 74.6% for N1b, 69.7% for N2a, 56.8% for N2b, and 63.4% for the VI groups. The DFS in the VI group was significantly worse than that in the N1b group (*p* = 0.003) but showed no significant differences compared to the N2a and N2b groups (*p* = 0.126 and *p* = 0.276; Figure 1A). The 5-year OS rates were 91.7% for N0, 91.3% for N1a, 84.9% for N1b, 83.0% for N2a, 76.1% for N2b, and 76.6% for the VI groups. The OS in the VI group was significantly worse than that in the N1b and N2a groups (*p* = 0.004 and *p* = 0.047) although the OS was not different from that in the N2b group (*p* = 0.906; Figure 1B).

### 3.5. New TNV Staging System Including VI

We devised a new TNV staging system by adding the presence of VI as the V stage into the traditional TN staging system: V0 and V1 were defined as the absence and presence of VI, respectively. Based on the oncological outcomes identified in the analysis, we considered V1 as N2a in TNV staging (summarized in Table 5). In the TNV staging system, the stages of V0 patients without VI were unchanged when compared to the conventional TN staging system. Furthermore, the staging of patients with N2a and N2b stages did not change, regardless of the V stage. However, T3N0V1 (*n* = 43) was upstaged from stage IIA to IIIB, T4aN0V1 (*n* = 11) from stage IIB to IIIC, T4bN0V1 (*n* = 4) from stage IIC to IIIC, T2N1V1 (*n* = 4) from stage IIIA to IIIB, and T4aN1V1 (*n* = 16) from stage IIIB to IIIC. In TNV staging, a total of 78 patients (3.5%) were upstaged, and the staging of 58 patients (5.6%) changed from stage II to stage III.

**Figure 2:**
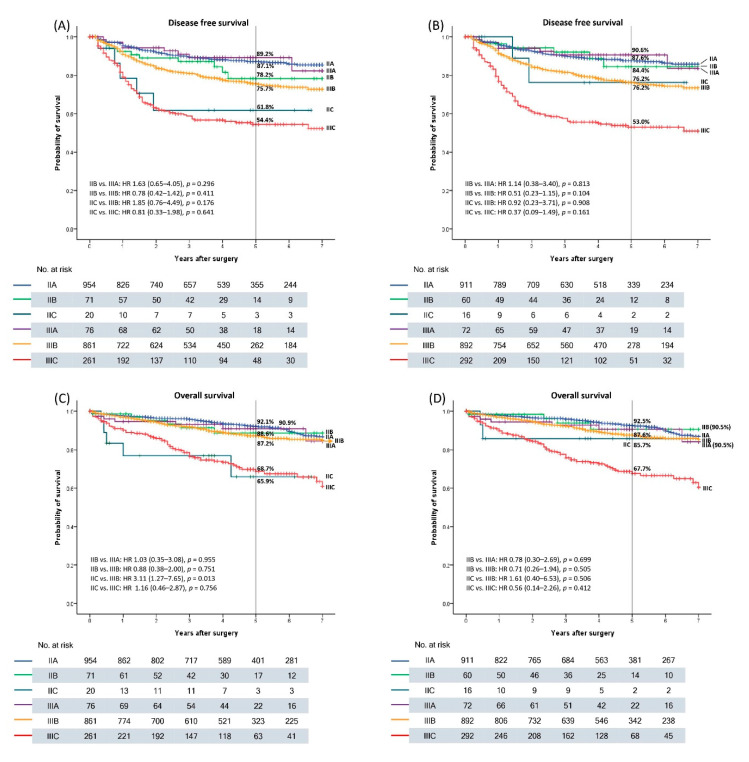
Kaplan–Meier curve in stage II–III colon cancer. (**A**) Disease-free survival (DFS) in TN stage. (**B**) DFS in new TNV stage. (**C**) Overall survival (OS) in TN stage. (**D**) OS in new TNV stage.

In Figure 2, the DFS and OS are shown according to the TN and TNV staging. The 5-year DFS rates were 87.1%, 78.2%, 61.8%, 89.2%, 75.7%, and 54.4% for IIA, IIB, IIC, IIIA, IIIB, and IIIC, respectively, in TN staging and 87.6%, 84.4%, 76.2%, 90.6%, 76.2%, and 53.0% for IIA, IIB, IIC, IIIA, IIIB, and IIIC in TNV staging, respectively (Figure 2A) and (B)). The 5-year OS rates were 92.1%, 88.6%, 65.9%, 90.9%, 87.2%, and 68.7% for IIA, IIB, IIC, IIIA, IIIB, and IIIC in TN staging, respectively, and 92.5%, 90.5%, 85.7%, 90.5%, 87.6%, and 67.7% for IIA, IIB, IIC, IIIA, IIIB, and IIIC in TNV staging, respectively (Figure 2C) and (D)). The OS in IIC was significantly worse than that in IIIB within the TN staging (*p* = 0.013), whereas the OS in IIC was similar to that of IIIB within the TNV staging system (*p* = 0.506).

## 4. Discussion

At present, TNM staging is considered an unrivalled system to predict prognosis in colon cancer. From the perspective of tumor cell dissemination, TNM staging consists of the starting point, the metastatic pathway, and the destination point of tumor cells. The starting point is T stage, defined as the depth of invasion of the primary tumor; the metastatic pathway is N stage, defined as regional LNM within the lymphatic system, and the destination point is M stage, defined as distant metastasis. Distant metastasis occurs through vascular and lymphatic channels, but LNM is an exclusive factor that determines stage III disease, and factors associated with the vascular system that are not included in the TNM stage [10,11,12,13,14].

Oncological outcomes do not always correspond to the TNM system. A national cohort study that evaluated oncological outcomes according to TNM staging showed that OS deteriorated in the order of I, IIIA, IIA, IIB, IIIB, IIC, and IIIC [19]. Another large cohort study demonstrated that T4aN0, classified as stage IIB, had a worse prognosis than T1-2N1 classified as stage IIIA, and T4bN0 classified as stage IIC had a lower 5-year OS rate than T3N1 classified as stage IIIB [20]. Similar oncological outcomes were observed in this study. The 5-year DFS rate was 78.2% and 61.8% in stage IIB and IIC and 89.2% and 75.7% in stage IIIA and IIIB, respectively. The 5-year OS rates were 88.6% and 65.9% in stages IIB and IIC and 90.9% and 87.2% in stages IIIA and IIIB, respectively. Inaccurate prognosis prediction based on the conventional TNM stage suggests that T4 tumor is a worse prognostic factor than the N1 stage. In this study, T4 tumors showed a stronger correlation with VI than with any N stage. Therefore, including VI into the TNM staging system could facilitate the alleviation of the discordance of oncological outcomes of the TNM staging system. 

In this study, we observed that the prognosis of patients in the VI group was significantly worse than that of patients in the N1 group. Moreover, OS was significantly worse in the VI group than in the N2a group. The results suggest that patients with VI had similar oncological outcomes to those with N2 stage without VI.

A few studies have proposed a new prognostic tool that upgrades TNM staging [21,22]. In a study that investigated a proposed a P-TNM staging system that combined the AJCC TNM staging with P-stage, consisting of the patient’s age, tumor grade, and tumor size, the results showed that P-TNM staging could improve prognostic prediction and clinical management in colon cancer [21]. Another study showed that incorporating microsatellite instability (MSI), BRAFV600E, and KRAS mutation status into the OS model through TNM staging improves the ability to precisely prognosticate in stages II and III colon cancer [22]. 

This study has several different aspects from those reported in previous studies. New TNVM staging devised in the present study is simple because we combined only VI with the conventional TNM staging system. Moreover, VI is a factor that is associated with the vascular system, which is one of the major metastatic pathways in colon cancer. In this novel TNVM staging system, the fact that V1 was considered as the N2 stage resulted in the upgrading of five sub-stages (T3N0V1, from IIA to IIIB; T4aN0V1, from IIB to IIIC; T4b N0V1, from IIC to IIIB; T2N1V1, from IIIA to IIIB; and T4aN1V1, from IIIB to IIIC). This result was observed for 78 of the 2243 patients, and they account for only 3.47% of the entire study population; moreover, 58 patients with stage II in the conventional TN staging were upgraded to stage III in the new TNV staging, accounting for only 5.55% of stage II patients in the conventional TN staging. Although only a small population of patients underwent stage change using the new system, even such a small change yielded significant improvement in the accuracy of predicting prognosis of patients with advanced diseases. Using the conventional TN staging system, patients with stage IIC showed worse 5- year DFS (61.8%) than those with IIIB (75.7%). However, as patients with T4N0V1 were now included in IIIC using the revised TNV system, the 5-year DFS of IIC patients were drastically improved to 76.2% as opposed to 61.8%, and the long-term outcome was comparable to those with IIIB. Therefore, we believe that the new system not only improves the accuracy of prognostic prediction, albeit in limited population, but also contributes to strengthen the conventional system by allocating patients in a more accurate stage group. 

This study had some limitations. First, the detection rate of VI was low in this study (10.0%). Recent studies have reported values ranging from 19% to 34% [15,17,23,24,25,26]. This difference may have been caused by the staining method. One study found that the detection rate of VI increased from 15.1% in the original pathological report, when using H-E staining, to 48.4%, when using an elastin stain. During the study period, all institutions that participated in this study used H-E staining for the detection of VI, which would result in a low VI detection rate [27]. This may be the reason why only a small population underwent stage change using the new staging system. Therefore, we expect that the use of elastin staining will increase the number of patients who will benefit from our new staging system, which includes VI as a parameter for staging. All institutions participating in the present study have been using elastin staining as a method to detect VI since 2017, but the follow-up period of those patients was too short to analyze their survival outcomes. Second, we did not distinguish between extramural VI (EMVI) and intramural VI (IMVI). Several studies have shown that EMVI is a stronger prognostic factor than IMVI in colon cancer [15,25]. Therefore, using elastin staining and distinguishing EMVI from IMVI would clarify the prognostic significance of VI. Finally, as this study had a retrospective design, the possibility of an inherent and unintentional selection bias cannot be ruled out. Thus, a large prospective cohort study using elastin staining would help validate the proposed TNVM staging system; a serial study is currently being conducted. 

## 5. Conclusions

In conclusion, VI had a similar prognostic predictive effect as that of the N2 stage without VI in stage II-III colon cancer. The findings from this study support the incorporation of the V-stage into the conventional TNM staging system for improved prediction of prognosis.

## Figures and Tables

**Table 1:** Baseline characteristics according to vascular invasion (VI) status.

Variables	Total Patients (*n* = 2243)	No VI (*n* = 2018)	VI (*n* = 225)	*p*
Age ≥ 65 years	1129(50.3)	1014(50.2)	115(51.1)	0.806
Sex, male	1238(55.2)	1105(54.8)	133(59.1)	0.213
ASA score ≥ 3	161(7.2)	146(7.2)	15(6.7)	0.754
Tumor location				0.379
Right colon	848(37.8)	769(38.1)	79(35.1)	
Left colon	1395(62.2)	1249(61.9)	146(64.9)	
Surgical approach				0.355
Laparoscopic	1412(63.0)	1264(62.6)	148(65.8)	
Conventional	831(37.0)	754(37.4)	77(34.2)	
T stage				<0.001
1–3	1951(87.0)	1795(88.9)	156(69.3)	
4	292(13.0)	223(11.1)	69(30.7)	
N stage				<0.001
0	1045(46.6)	987(48.9)	58(25.8)	
1	762(34.0)	696(34.5)	66(29.3)	
2	436(19.4)	335(16.6)	101(44.9)	
TNM stage				<0.001
II	1045(46.6)	987(48.9)	58(25.8)	
III	1198(53.4)	1031(51.1)	167(74.2)	
Number of LN harvest < 12	369(16.5)	336(16.7)	33(14.7)	0.447
Histological grade, poor	210(9.4)	180(8.9)	30(13.3)	0.031
Lymphatic invasion, yes	1067(47.6)	909(45.0)	158(70.2)	<0.001
Perineural invasion, yes	567(25.3)	468(23.2)	99(44.0)	<0.001
Adjuvant chemotherapy, yes	1772(79.0)	1586(78.6)	186(82.7)	0.155
Recurrence, yes	420(18.7)	349(17.3)	71(31.6)	<0.001
Local recurrence, yes	51 (2.3)	46 (2.3)	5 (2.2)	0.956

VI, vascular invasion; ASA, American Society of Anesthesiologist; T, tumor; N, node; TNM, tumor node metastasis; LN, lymph node. Proportion ( ) are presented for categorical data.

**Table 2:** Patient’s clinicopathological characteristics according to the study groups.

Variables	N0 (*n* = 987)	N1 (*n* = 696)	N2 (*n* = 335)	VI (*n* = 225)	*p* *	*p* **
Age ≥ 65 years	504(51.1)	349(50.1)	161(48.1)	115(51.1)	0.809	0.479
Sex, male	571(57.9)	359(51.6)	175(52.2)	133(59.1)	0.028	0.109
ASA score ≥ 3	70(7.1)	59(8.5)	17(5.1)	15(6.7)	0.252	0.426
Tumor location					0.351	0.964
Right side	391(39.6)	261(37.5)	117(34.9)	79(35.1)		
Left side	596(60.4)	435(62.5)	218(65.1)	146(64.9)		
Surgical approach					0.707	0.303
Laparoscopic	615(62.3)	433(63.6)	206(61.5)	148(65.8)		
Conventional	372(37.7)	253(36.4)	129(38.5)	77(34.2)		
T stage					<0.001	0.003
1–3	911(92.3)	615(88.4)	269(80.3)	156(69.3)		
4	76(7.7)	81(11.6)	66(19.7)	69(30.7)		
N stage						<0.001
0	987(100.0)			58(25.8)		
1		696(100.0)		66(29.3)		
2			335(100.0)	101(44.9)		
TNM stage						<0.001
II	987(100.0)	0	0	58(25.8)		
III	0	696(100.0)	335(100.0)	167(74.2)		
Number of LN harvest < 12	173(17.5)	133(19.1)	30(9.0)	33(14.7)	<0.001	0.036
Histological grade, poor	59(6.0)	67(9.6)	54(16.1)	30(13.3)	<0.001	0.365
Lymphatic invasion, yes	154(15.6)	493(70.8)	262(78.2)	158(70.2)	<0.001	0.032
Perineural invasion, yes	165(16.7)	167(24.0)	136(40.6)	99(44.0)	<0.001	0.424
Adjuvant chemotherapy, yes	696(70.5)	595(85.5)	295(88.1)	186(82.7)	<0.001	0.072
Recurrence, yes	107(10.8)	136(19.5)	106(31.6)	71(31.6)	<0.001	0.983
Local recurrence, yes	16(1.6)	15(2.2)	15(4.5)	5(2.2)	0.026	0.159

VI, vascular invasion; ASA, American Society of Anesthesiologist; T, tumor; TNM, tumor node metastasis; LN, lymph node. * *p*-value comparing all groups. ** *p*-value comparing N2 and VI groups. Proportion ( ) are presented for categorical data.

**Table 3:** Disease-free survival: the univariate and multivariate analyses.

	Univariate Analysis		Multivariate Analysis	
Variables	HR (95% CI)	*p*	HR (95% CI)	*p*
Age ≥ 65 years	1.258 (1.039–1.524)	0.019	1.260 (1.039–1.527)	0.019
Sex, male	1.099 (0.906–1.333)	0.340		
ASA score ≥ 3	1.323 (0.918–1.908)	0.134		
Tumor location				
Right side	Reference			
Left side	1.198 (0.979–1.467)	0.080		
Surgical approach				
Laparoscopic	Reference		Reference	
Conventional	1.318 (1.086–1.599)	0.005	1.307 (1.073–1.593)	0.008
T stage				
1–3	Reference		Reference	
4	2.426 (1.931–3.048)	<0.001	1.920 (1.511–2.439)	<0.001
N stage				
0	Reference			
1	1.785 (1.410–2.261)	<0.001		
2	3.147 (2.472–4.006)	<0.001		
TNM stage				
II	Reference			
III	2.248 (1.822–2.772)	<0.001		
Number of LN harvest < 12	1.275 (1.001–1.625)	0.049	1.453 (1.132–1.864)	0.003
Histological grade, poor *	1.627 (1.220–2.171)	0.001	1.315 (0.977–1.770)	0.070
VI, yes	2.149 (1.665–2.774)	<0.001		
Lymphatic invasion, yes	1.602 (1.320–1.944)	<0.001	0.882 (0.699–1.114)	0.291
Perineural invasion, yes	1.557 (1.271–1.907)	<0.001	1.259 (1.015–1.561)	0.036
Adjuvant chemotherapy, no	0.909 (0.696–1.186)	0.480		
Group				
N0	0.538(0.418–0.693)	<0.001	0.543 (0.407–0.722)	<0.001
N1	Reference		Reference	
N2	1.778(1.379–2.292)	<0.001	1.738 (1.343–2.251)	<0.001
VI	1.904(1.429–2.537)	<0.001	1.704 (1.267–2.291)	<0.001

HR, hazard ratio; CI, confidence interval; ASA, American Society of Anesthesiologist; T, tumor; N, node; TNM, tumor node metastasis; LN, lymph node; VI, vascular invasion. * Poorly differentiated adenocarcinoma, mucinous carcinoma, signet-ring cell carcinoma.

**Table 4:** Overall survival: the univariate and multivariate analyses.

	Univariate Analysis		Multivariate Analysis	
Variables	HR (95% CI)	*p*	HR (95% CI)	*p*
Age ≥ 65 years	1.650 (1.290–2.112)	<0.001	1.282 (0.990–1.661)	0.060
Sex, male	1.349 (1.051–1.732)	0.019	1.319 (1.026–1.696)	0.031
ASA score ≥ 3	2.921 (2.077–4.107)	<0.001	2.531 (1.774–3.611)	<0.001
Tumor location				
Right side	Reference			
Left side	1.056 (0.821–1.359)	0.671		
Surgical approach				
Laparoscopic	Reference		Reference	
Conventional	1.711 (1.341–2.184)	<0.001	1.569 (1.225–2.011)	<0.001
T stage				
1–3	Reference		Reference	
4	2.726 (2.057–3.612)	<0.001	2.110 (1.567–2.842)	<0.001
N stage				
0	Reference			
1	1.367 (1.016–1.840)	0.039		
2	2.642 (1.964–3.555)	<0.001		
TNM stage				
II	Reference			
III	1.805 (1.396–2.333)	<0.001		
Number of LN harvest < 12	1.507 (1.127–2.014)	0.006	1.588 (1.177–2.142)	0.002
Histological grade, poor *	1.812 (1.279–2.566)	0.001	1.577 (1.095–2.271)	0.014
VI, yes	2.260 (1.652–3.092)	<0.001		
Lymphatic invasion, yes	1.607 (1.256–2.056)	<0.001	1.251 (0.919–1.704)	0.154
Perineural invasion, yes	1.219 (0.933–1.594)	0.147		
Adjuvant chemotherapy, no	2.055 (1.569–2.692)	<0.001	2.268 (1.699–3.027)	<0.001
Group				
N0	0.785 (0.573–1.076)	0.132	0.870 (0.598–1.266)	0.467
N1	Reference		Reference	
N2	1.790 (1.268–2.528)	0.001	1.949 (1.366–2.779)	<0.001
VI	2.295 (1.594–3.304)	<0.001	2.301 (1.582–3.348)	<0.001

HR, hazard ratio; CI, confidence interval; ASA, American Society of Anesthesiologist; T, tumor; N, node; TNM, tumor node metastasis; LN, lymph node; VI, vascular invasion. * Poorly differentiated adenocarcinoma, mucinous carcinoma, signet-ring cell carcinoma.

**Table 5:** The details of TN stage and TNV stage in stage II-III colon cancer.

T	N	V	TN Stage	TNV Stage	Change	N (%)
T3	N0	V0	IIA	IIA	No	911 (40.6)
		V1	IIA	IIIB	Yes	43 (1.9)
T4a	N0	V0	IIB	IIB	No	60 (2.7)
		V1	IIB	IIIC	Yes	11 (0.5)
T4b	N0	V0	IIC	IIC	No	16 (0.7)
		V1	IIC	IIIC	Yes	4 (0.2)
T1	N1	V0	IIIA	IIIA	No	22 (1.0)
		V1	IIIA	IIIA	No	1 (0.0)
T2	N1	V0	IIIA	IIIA	No	47 (2.1)
		V1	IIIA	IIIB	Yes	4 (0.2)
T3	N1	V0	IIIB	IIIB	No	546 (24.3)
		V1	IIIB	IIIB	No	44 (2.0)
T4a	N1	V0	IIIB	IIIB	No	70 (3.1)
		V1	IIIB	IIIC	Yes	16 (0.7)
T4b	N1	V0	IIIC	IIIC	No	11 (0.5)
		V1	IIIC	IIIC	No	1 (0.0)
T1	N2a	V0	IIIA	IIIA	No	2 (0.1)
		V1	IIIA	IIIA	No	0 (0.0)
T2	N2a	V0	IIIB	IIIB	No	5 (0.2)
		V1	IIIB	IIIB	No	0 (0.0)
T3	N2a	V0	IIIB	IIIB	No	154 (6.9)
		V1	IIIB	IIIB	No	23 (1.0)
T4	N2a	V0	IIIC	IIIC	No	39 (1.7)
		V1	IIIC	IIIC	No	12 (0.5)
T1	N2b	V0	IIIB	IIIB	No	0 (0.0)
		V1	IIIB	IIIB	No	1 (0.0)
T2	N2b	V0	IIIB	IIIB	No	0 (0.0)
		V1	IIIB	IIIB	No	2 (0.1)
T3	N2b	V0	IIIC	IIIC	No	106 (4.7)
		V1	IIIC	IIIC	No	40 (1.8)
T4	N2b	V0	IIIC	IIIC	No	27 (1.2)
		V1	IIIC	IIIC	No	25 (1.1)

T, tumor; N, node; V, vascular invasion.

## Data Availability

Not applicable.

## Supplementary Materials

The following are available online at https://www.mdpi.com/article/10.3390/biomedicines9080888/s1, Table S1: Results of multiple logistic regression analysis of the factors related to vascular invasion.
