# Supplementary material for: Addition of V-Stage to Conventional TNM Staging to Create the TNVM Staging System for Accurate Prediction of Prognosis in Colon Cancer: A Multi-Institutional Retrospective Cohort Study
Source: Biomedicines. 2021 Jul 25;9(8):888. doi: 10.3390/biomedicines9080888 (PMC8389700; doi:10.3390/biomedicines9080888)
Supplement: Supplementary file 1 [file biomedicines-09-00888-s001.zip › biomedicines-1313598-supplementary.pdf]

Supplementary Material

Table S1. Results of multiple logistic regression analysis of the factors related to vascular invasion.

**Variables**	**OR (95% CI)**	***p*-Value**
T4 tumor	2.516 (1.794–3.527)	<0.001
N stage		
N0	Reference	
N1	1.106 (0.726–1.684)	0.639
N2	2.873 (1.893–4.361)	<0.001
Histological grade, poor	0.955 (0.608–1.498)	0.840
Lymphatic invasion, yes	1.659 (1.145–2.403)	0.007
Perineural invasion, yes	1.706 (1.258–2.314)	0.001

OR, odds ratio; CI confidence interval.
